# Oxidative stress and senescence in aging kidneys: the protective role of SIRT1

**DOI:** 10.17179/excli2024-7519

**Published:** 2024-08-27

**Authors:** Waleed Hassan Almalki, Salem Salman Almujri

**Affiliations:** 1 Department of Pharmacology, College of Pharmacy, Umm Al-Qura University, Makkah, Saudi Arabia; 2 Department of Pharmacology, College of Pharmacy, King Khalid University, Abha 61421, Aseer, Saudi Arabia

**Keywords:** SIRT1, SASP, oxidative stress, cellular senescence, homeostasis

## Abstract

Aging leads to a gradual decline in kidney function, making the kidneys increasingly vulnerable to various diseases. Oxidative stress, together with cellular senescence, has been established as paramount in promoting the aging process of the kidney. Oxidative stress, defined as an imbalance between ROS formation and antioxidant defense mechanisms, has been implicated in the kidney's cellular injury, inflammation, and premature senescence. Concurrently, the accumulation of SCs in the kidney also exacerbates oxidative stress via the secretion of pro-inflammatory and tissue-damaging factors as the senescence-associated secretory phenotype (SASP). Recently, SIRT1, a nicotinamide adenine dinucleotide (NAD)-dependent deacetylase, has been pivotal in combating oxidative stress and cellular senescence in the aging kidney. SIRT1 acts as a potential antioxidant molecule through myriad pathways that influence diverse transcription factors and enzymes essential in maintaining redox homeostasis. SIRT1 promotes longevity and renal health by modulating the acetylation of cell cycle and senescence pathways. This review covers the complex relationship between oxidative stress and cellular senescence in the aging kidney, emphasizing the protective role of SIRT1.

See also the graphical abstract(Fig. 1).

## Introduction

Aging is a complex process characterized by progressive physiological declines in multiple organs, including the kidney (Fan et al., 2024[104]; Glassock and Rule, 2016[126]). The kidneys act to maintain homeostasis by filtering metabolic waste products, regulating electrolyte and fluid balance, and controlling blood pressure (Li and Wang, 2018[206]). However, as one age, kidney function gradually declines, signaling renal aging or age-associated kidney dysfunction (Minami et al., 2023[238]; Yang et al., 2019[375]). On a structural level, nephrons, the units of the kidney, are lost and responsible for reducing renal mass (Kimura et al., 2017[186]). This nephron loss is associated with glomerulosclerosis, or the hardening of the glomeruli, and tubulointerstitial fibrosis, characterized by an increased connective tissue deposition in the kidney interstitial (Hussain et al., 2024[155]; Tang et al., 2020[317]). These alterations explain the reduction in GFR, an important parameter for the kidney (Denic et al., 2022[91]; Qiu et al., 2018[280]). In a simplified format, functionally, old kidneys poorly conserve sodium and water, cannot concentrate urine to full potential, and do not respond as robustly to various metabolic hormone regulation (Cailleaux and Cohen-Solal, 2022[59]). Such modifications increase older people's susceptibility to dehydration, electrolyte disturbances and high blood pressure (Denic et al., 2016[90]; Satturwar and Parwani, 2023[296]). Older individuals are also highly vulnerable to more acute types of kidney injury, such as AKI/CKD, as aging kidneys have a poorer ability to repair following acute injury (Fang et al., 2020[107]; Shen et al., 2024[302]). Various mechanisms at a cellular level in the kidney drive the aging process, such as oxidative stress, cellular senescence, and inflammation (Epstein, 1996[100]). Oxidative stress arises when an increased ROS production exceeds the capacity of the antioxidant defense system, leading to cellular damage (Bridges and Zalups, 2017[56]). The irrecoverable cell growth inhibition causes cellular senescence, tissue damage and fibrosis. Additionally, inflammation contributes to both the progression of renal injury and fibrosis (Aaseth et al., 2021[1]; Chen et al., 2023[71]).

### Role of oxidative stress and senescence in age-related kidney dysfunction

Oxidative stress and cellular senescence are the major factors involved in the progression of age-related changes in the kidney (Gekle, 2017[122]). Oxidative stress results from an imbalance between the production of potentially hazardous ROS and the body's antioxidant defenses, leading to oxidative damage to cellular proteins, lipids, and DNA (Bahl et al., 2024[23]; Sobamowo and Prabhakar, 2017[311]). This oxidative damage further exacerbates the deterioration of renal function by inducing inflammation, fibrosis and apoptosis of renal cells in aging kidneys (Hu et al., 2023[149]; Shankland et al., 2021[300]). Senescent cells, which have irreversibly exited the cell cycle, are another important contributor to renal aging (Chen et al., 2019[69]). Cellular senescence is associated with a secretion of proinflammatory cytokines, growth factors, and proteases collectively known as the SASP that eventually leads to accumulation of senescent cells in the kidney with age (Hommos et al., 2017[146]). SASP promotes tissue inflammation and fibrosis, aggravating renal injury. Therefore, oxidative stress and senescence form a vicious cycle in the aging kidney (Frassetto et al., 2020[116]; Zhu et al., 2024[396]). Cellular senescence is a new attractive target for both anti-oxidative stress treatment and anti-inflammatory therapy, whereas these therapies directly or indirectly converge to each other due to oxidative stress directly induces senescence, and senescence secretes proinflammatory cytokines to exaggerate oxidative damage (Akasaka-Manya et al., 2016[5]; Modi et al., 2024[241]). This dynamic promotes renal structural and functional impairment as aging progresses and may further aggravate CKD and other age-related renal diseases (Cheng et al., 2024[76]; Rodríguez-Castro and Córdova, 2011[285]).

## Oxidative Stress and Kidney Aging

### Reactive oxygen species (ROS) and oxidative stress

ROS is always naturally formed, even in cellular pathways (Wakino and Itoh, 2016[349]). They are important fuels of aerobic organisms as they are produced as by-products of aerobic metabolism in numerous cell organelles, such as mitochondria, chloroplasts, peroxisomes, cytosol and plasma membrane (Lyulcheva-Bennett et al., 2023[226]; Mir et al., 2024[239]; Musso et al., 2016[249]). ROS are produced when O_2 _acquire one electron at a time, forming reactive species, such as O_2_, H_2_O_2_, and OH (van den Anker et al., 2018[340]). ROS have dual roles in cells based on their concentrations (Alharbi et al., 2022[8]; Ferenbach and Bonventre, 2015[110]). At high enough levels, they will tend to react around everything. They can lead to oxidative damage to proteomics, DNA/RNA, and lipids and, in turn, cellular dysfunction or even death (Alharbi et al., 2022[9]; Kanasaki et al., 2012[170]). Lower concentrations of ROS, in contrast, can play positive roles, regulating cellular signaling pathways and modulating gene expression (Kooman et al., 2015[192]). Red light can be used to prevent oxidative stress, which occurs when the generation of ROS exceeds the reducing capabilities of the cell, in which free radicals are not generated in very high numbers as a result of an imbalance state of redox (Alharbi et al., 2021[10]; Morigi et al., 2018[245]; Zheng et al., 2022[392]). This imbalance can attack cellular components and lead to different diseases, including varicocele, in which ROS formation is responsible for testicular tissue damage and decreased fertility (Bauer et al., 1991[31]). In this regard, we have determined that the leptin-stimulated generation of ROS in cultured SMCs from obese-hyperleptinemic rats is partially resistant in this context of leptin resistance (Karam and Tuazon, 2013[172]). These changes were accompanied by higher protein expression of p22phox and gp91phox subunits of the NADPH oxidase complex and protein markers of leptin resistance, i.e. pSTAT3, PTP1B and SOCS3 (Alharbi et al., 2022[11]; Huber et al., 2012[152]). These studies suggest that ROS may play a critical role in several other physiological processes, including brain oxidative stress during most likely hypotensive sepsis, but are ameliorated with simvastatin pre-treatment (Bouarich et al., 2021[52]). In this regard, knowledge of ROS generation and consequent oxidative stress is important for developing treatments that can limit their negative effects, and help the maintenance of cell health (Alvis and Hughes, 2015[13]; Bhat et al., 2024[41]).

### Sources of ROS in aging kidneys

Sources of ROS in aging kidneys are multifactorial and linked to different cellular processes (Bitzer and Wiggins, 2016[48]). XD enzyme complex serves as a major source of ROS that oxidizes hypoxanthine to form xanthine and produces uric acid (Bhat et al., 2022[44]; Zhou et al., 2008[394]). XDH is converted to XOD in aging kidneys, inferring more ROS generation (Lin et al., 2022[211]). AGEs, generated by non-enzymatic glycation and accumulate with aging in tissues, represent another major source of ROS (Bhat et al., 2024[45]; Sato and Yanagita, 2018[294]; Zhang et al., 2023[387]). AGEs trigger the RAGE to increase oxidative stress, causing ROS generation by NADPH oxidase in mitochondria (Choudhury and Levi, 2011[77]). In aging kidneys, mitochondrial dysfunction is also an important factor in ROS generation (Vlassara et al., 2009[345]). Increased ROS production increases oxidative damage at the cellular level due to mitochondrial dysfunction (Baylis, 2012[33]). This area of investigation assesses the regulation of renal mitochondrial bioenergetics and ROS generative pathways by sex hormones (Bhat et al., 2023[46]; Perico et al., 2024[270]). Estrogen, although not excellent for all renal diseases, is generally considered a protective agent, and its depletion can potentially accelerate the deterioration of renal mitochondrial function and enhance ROS production (Baylis, 2005[33]). The aging-related sources of ROS in kidneys are diverse but result from multiple cellular mechanisms (Bolignano et al., 2014[50]). It is important to understand these mechanisms to develop therapeutic strategies to protect and not damage kidney integrity in aging by producing effective elimination and maintaining healthy cells, reducing/abolishing proteinuria through protection against injury, and maintaining cell integrity and homeostasis under sublethal requirement oxidative stress with ROS, insignificant injury (Gupta et al., 2021[134]; Kaysen and Myers, 1985[176]).

### Consequences of oxidative stress on kidney function

Aging-aged human kidneys are affected by oxidative stress (Wang et al., 2014[357]). Oxidative stress, the imbalance between ROS production and its scavenging by antioxidant defenses, results in peroxidation of lipids, damage to DNA, and oxidation of proteins (Pannarale et al., 2010[265]; Tufail et al., 2024[333]). The harm is done to proper cellular function and becomes a key pathogenic mechanism of many kidney diseases (Hayashi et al., 2019[140]; Hussain et al., 2023[153]). One major hormonal effect of oxidative stress in the kidneys is inflammation. ROS stimulate inflammatory signaling pathways, releasing chemotactic signals such as cytokines and other pro-inflammatory cytokines to attract immune cells to the injured region (Jo et al., 2023[167]; Uddin et al., 2021[335]). This further leads to progressive glomerulosclerosis, tubulointerstitial fibrosis, and excessive extracellular matrix deposition in the renal interstitium (Wei et al., 2020[359]). These two illnesses limit the filterability of the kidneys, and subsequently, the GFR goes down (Dybiec et al., 2022[99]; Hussain et al., 2023[154]). In addition, oxidative stress can lead renal cells to apoptosis (Hu et al., 2020[148]). Losing a portion of the cells that perform specific functions further impairs the kidney's ability to maintain homeostasis - the balance that your body requires to process fluids, electrolyte/salt regulation, and waste disposal (Nguyen et al., 2018[254]). Furthermore, oxidative stress can reduce the ability of renal cells to regenerate, thus rendering the kidneys less responsive to acute injury recovery (Gava et al., 2011[121]; Rohilla et al., 2023[286]).

## Role of Epigenetic Processes in Regulating SIRT1 Expression

Epigenetic mechanisms greatly contribute to the regulation of gene expression independently from changes in DNA sequence (Alves-Fernandes and Jasiulionis, 2019[12]). These mechanisms involve DNA methylation, histone modifications, and the role of non-coding RNAs acting to modulate expression levels of important homeostatic genes such as SIRT1 (Ashapkin et al., 2017[18]). On the other hand, DNA methylation is one of the common epigenetic modifications that involves adding a methyl group to cytosine residues at position 5 nucleotide and has an important function as a gene silencing process (Ayissi et al., 2014[20]). Hypermethylation in its promoter region can lead to transcriptional silencing, while hypomethylation could promote its expression. Moreover, the methylation of the SIRT 1 promoter in response to environmental stresses such as oxidative stress may affect expression levels (D'Onofrio et al., 2018[97]; Thapa et al., 2024[323]). The changes in DNA methylation in the SIRT1 promoter region were related to the increased expression level and activity of SIRT1 during a stress reaction. Histone proteins possess several post-translational modifications, such as acetylation and methylation, phosphorylation, and ubiquitination (Fernandes et al., 2017[111]). These modifications alter chromatin structure and may influence gene expression. Specifically, histone acetylation, commonly linked to active gene expression, occurs when the acetyl group is transferred to lysine residues of histone tails (Fledderus et al., 2021[112]). This formation reduces affinity between DNA and histone tails, making chromatin more open and relaxed. It can also condense chromatin by working as a target gene inhibitor. SIRT1 is that type of inhibitor (Garcia-Peterson and Li, 2021[120]; Thapa et al., 2023[325]). This protein serves as an NAD + - dependent deactylase, meaning it removes acetyl groups from histones. Sirtuin 1 deacetylates histones, leading to chromatin compaction and target gene transcriptional inhibition (Jia et al., 2019[163]).

On the contrary, SIRT1 expression levels may increase if the latest undergoes the same end. The situation is then that the SIRT1 promoter region will behave in the form of higher acetylation (Nakatani and Inagi, 2016[252]). These steps constitute a cycle that changes target gene expression depending on the internal cellular signals. MiRNAs and lncRNAs are two non-coding RNAs that regulate gene expression post-transcriptional stage. As is known, miRNAs are capable of binding to the 3'UTR of SIRT1 mRNA, which later leads to its degradation or translational repression.

On the other hand, some lncRNAs may act as a reservoir for miRNAs in the form of molecular sponges. Thus, these miRNAs will not interact with the SIRT1 mRNA sequence (Pyo et al., 2020[278]). If UTR is not bonded with miR-34a, it cannot destroy it. This situation will lead to mRNA stabilization that results in enhanced SIRT1 synthesis. The miRNAs and lncRNAs system create a highly balanced approach to SIRT1 expression levels (Tao et al., 2023[320]).

## Cellular Senescence in Aging Kidneys

### Essence and features of cellular senescence

Cellular senescence is a state of irreversible cell cycle arrest triggered by various stress stimuli such as DNA damage, oxidative stress, and oncogene activation (Islam et al., 2024[159]; Musso and Oreopoulos, 2011[250]; Thapa et al., 2023[327]). It protectively prevents the generation of cellulite and, in the end, helps to avoid the risk of hopefully getting malignant cells (Lonergan, 1988[222]). Nevertheless, senescent cells are far from inert, characterized by their idiosyncratic phenotype, which entails massive cellular morphological and functional alterations (Esposito and Dal Canton, 2010[102]). A large and flat morphology characterizes senescent cells, enhanced SA-β-gal activity, and production of a plethora of pro-inflammatory cytokines, chemokines, growth factors and proteases known as SASP (Sato and Yanagita, 2019[295]). In normal cells, the SASP can enhance the senescence growth arrest and modulate the tissue microenvironment; additionally, the SASP can provoke inflammation and tissue remodeling, thereby promoting aging and age-related pathologies (Buemi et al., 2005[57]). Such cells can be arrested or removed to the benefit of the tissue. Still, their presence *in vivo* mandates further research to learn how they are regulated during aging and to identify the causal factors that provoke them in human pathologies (Sands, 2012[292]).

### Mechanisms of senescence induction in kidney cells

Cellular senescence in kidney cells is induced by various types of stress and signaling pathways (Jassal and Oreopoulos, 1998[162]). Among these is damage to genetic material, especially DNA, frequently arising from oxidative stress (Baylis and Corman, 1998[34]). DNA damage is also the result of other factors; ROS are impermanent, high-reactive compounds that have the potential to produce harm in biological macromolecules such as DNA, proteins, and lipids and can initiate the DDR (Artioli et al., 2019[16]; Jiang et al., 2017[164]; Li et al., 2024[204]). DDR activation at this moment results in cell cycle arrest mainly through the p53/p21 and p16INK4a/Rb pathways, controlling important elements of senescence (Khan et al., 2017[178]; Wang et al., 2018[355]). It also turned out that oxidative stress was a potent inducer of senescence in kidney cells (Wang et al., 2021[358]). ROS helps to disturb cellular homeostasis and leads to oxidative damage and mitochondrial impairment (Franzin et al., 2020[115]; Presta et al., 2012[276]). Due to ROS and a vicious circle where ROS production is exacerbated by the loss of the ability to access functioning mitochondria, cellular aging and senescence may be accelerated (Schmitt et al., 2015[297]). Telomere shortening is another mechanism leading to senescence, which occurs through normal cellular replication (Nguyen and Goldfarb, 2012[256]). More importantly, critically short telomeres are considered DSB, which triggers DDR pathways. Senescence in kidney cells is also driven by inflammation. Sustained exposure to inflammatory cytokines and growth factors, as seen in chronic inflammatory conditions, can also promote senescence via paracrine signaling (Hirakawa et al., 2017[145]; Speeckaert et al., 2014[312]). This phenomenon is commonly called inflammaging, and it promotes the damage and senescence of tissues (Wiggins, 2009[363]).

#### Senescence induction and epigenetic changes 

Events such as histone methylation and acetylation that induce chromatin changes in gene expression profiles necessary for senescence-associated growth arrest and the SASP can also be modified by miRNAs (Han et al., 2022[137]; Kuro-o, 2010[195]). Understanding these mechanisms is important for developing targeted interventions to prevent or ameliorate renal senescence, preserving kidney function and delaying the onset of age-related kidney pathologies (Meng et al., 2020[231]).

#### Telomere shortening

Telomere shortening is a critical mechanism for cellular senescence and aging (Li et al., 2021[207]). Telomeres are sequences of repetitive nucleotides found at both ends of the chromosome and which function to protect the ends from degradation and fusion with the ends of other chromosomes (Mohamad Kamal et al., 2020[242]; Obas and Vasan, 2018[260]). During cell division, the telomeres are shortened because the DNA polymerases only partially replicate the DNA ends of the linear chromosomes, with each proliferation narrowing the telomeres (Liu, 2022[220]; Ogrodnik, 2021[261]). Like all somatic cells, renal cells encounter this programmed shortening. When this results in the shortest telomere in the cell, the cell recognizes that a critical length threshold affecting homologous recombination and generating significant loss of genetic material has been reached, and this magnifies a signal to DDR (Gonzalo et al., 2017[127]; Imran et al., 2021[158]; Jones et al., 2015[168]). Activation of DDR pathways causes cell-cycle arrest, mostly directed towards cellular senescence via p53/p21 and p16 INK4a/Rb pathways (Turner et al., 2019[334]; Zhu et al., 2019[398]). This is due to the secretion of proinflammatory cytokines, growth factors, and proteases from senescent cells, where declaring the SASP can potentially promote tissue dysfunction and inflammation (Aubert, 2014[19]; Rossiello et al., 2022[288]). This is particularly important when considering aging kidneys, which accelerate age-related renal function deterioration, predisposing an individual to CKD (Prasad et al., 2017[275]). Thus, uncovering the mechanisms regarding the effect of telomere shortening on kidney aging is important for designing therapies to maintain the telomere length and to protect the cells against senescence in elderly kidneys (Mikhelson and Gamaley, 2012[237]; Morgan et al., 2018[244]; Opresko and Shay, 2017[263]).

#### Oxidative stress-induced senescence

Oxidative stress-induced senescence is a key step in cellular aging and tissue dysfunction (Li et al., 2022[203]). ROS are produced as by-products of metabolic reactions or by exposure to external stimuli, and they can damage DNA, proteins, and lipids (Chandra and Rajawat, 2021[64]; Lin and Epel, 2022[210]). The activation of DDR pathways is one of the principle phenotypes whereby age-related DNA damage triggers cell cycle arrest via p53/p21 and p16INK4a/Rb signaling (Liu et al., 2023[217]; Rellmann et al., 2021[283]). In more chronic conditions, oxidative stress in kidney cells has been shown to increase senescence, leading to inflammation, fibrosis and reduced regenerative capacity (Pintó-Marijuan and Munné-Bosch, 2014[272]). This is detrimental to age-related renal decline and induces CKD susceptibility (Kaarniranta et al., 2018[169]). The aging kidney requires therapeutic strategies capable of preventing or at least reducing the progression of kidney disease, and the elucidation of the pathways involved in oxidative stress-induced premature senescence is essential for the development and success of such interventions (Nousis et al., 2023[259]; Terao et al., 2022[321]).

### Role of senescent cells in kidney aging and disease

Cells that have become senescent contribute to kidney aging and renal disease development (Wiggins, 2011[364]). Such accumulation in the kidneys over time is partly the result of aging factors - most notably oxidative stress, DNA damage and telomere shortening - and are cells that have undergone an irreversible cell cycle arrest (Kłoda et al., 2015[190]; Verbalis, 2014[343]). The differentiated cells enter a senescent state, and some acquire SASP, overproducing pro-inflammatory interleukins, chemokines, growth factors, and proteases (Denecke et al., 2015[89]; Smykiewicz et al., 2018[310]). The SASP drives transcriptomes for age-associated inflammation and promotes tissue remodeling, fibrosis, and widespread chronic inflammation, all dialing in a decreasing trajectory for kidney function with age (Fusco et al., 2016[118]). Senescent cells also limit kidney regenerative capacity, thus impeding normal kidney repair following injury (Fougeray and Pallet, 2015[114]). This further deteriorates age-induced renal insufficiency and then promotes vulnerability to CKD, such as glomerulosclerosis and tubulointerstitial fibrosis (Lamb et al., 2003[198]; Weide and Huber, 2011[360]). Therapeutic strategies targeting senescent cells and their deleterious effects may offer hope in maintaining kidney function and outcomes in the aging population (Bennett, 1990[36]; Musso et al., 2015[248]). 

#### Senescence-associated secretory phenotype (SASP)

**Figure 2:**
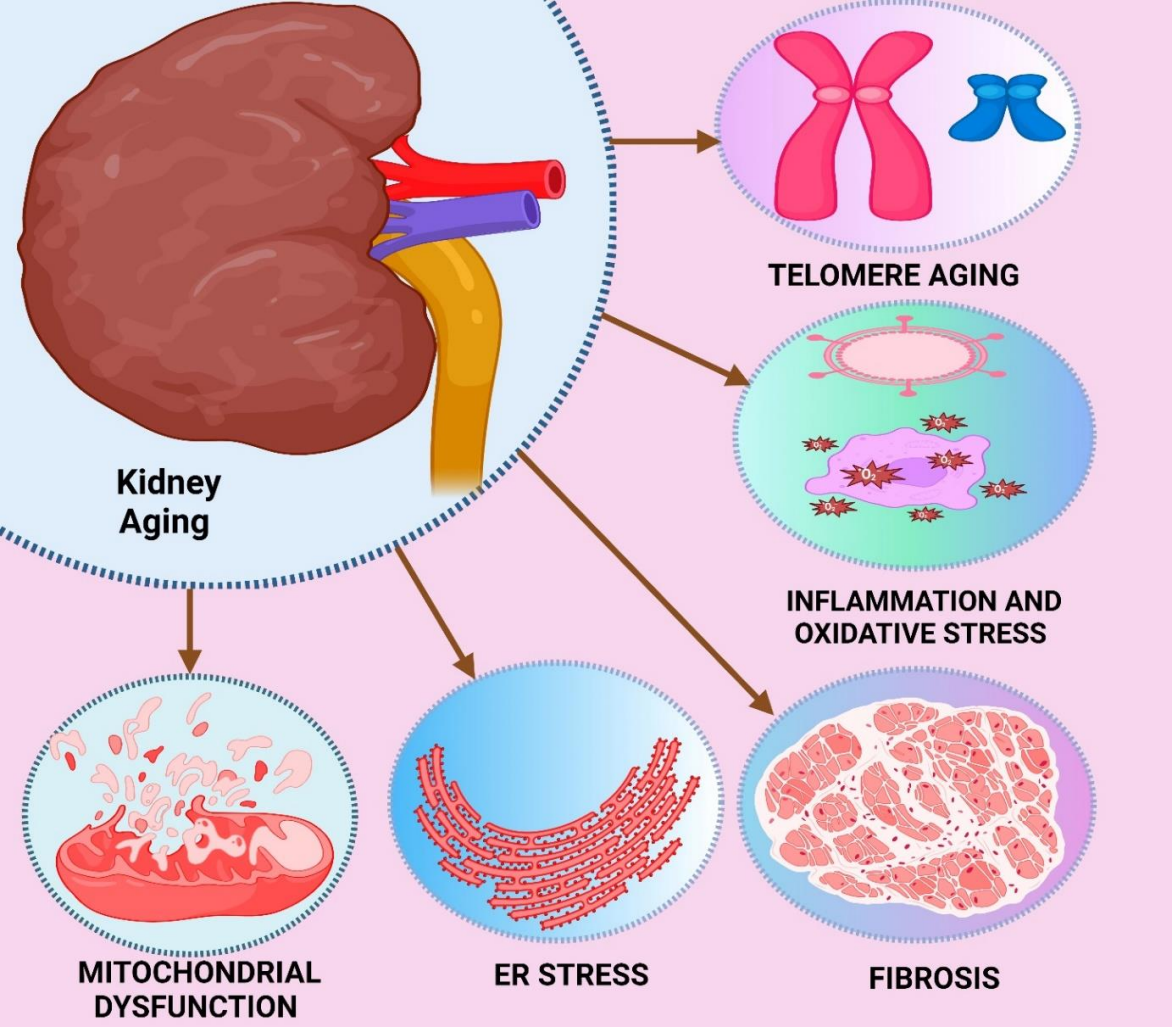
The image illustrates the process of kidney aging, highlighting key factors such as telomere aging, inflammation and oxidative stress, mitochondrial dysfunction, ER stress, and fibrosis. Each factor contributes to the overall decline in kidney function associated with aging.

Senescent cells exhibit a three-part phenotype called the SASP, comprising hundreds of pro-inflammatory cytokines and chemokines, growth factors, and proteases (Guebre-Egziabher et al., 2013[131]. This secretory phenotype has a major effect on the tissue microenvironment and a role in various age-related pathologies (Charloux et al., 2008[66]). The SASP is a double-edged singer in this narrative on kidney aging. First, it maintains the growth arrest of senescent cells, which prevents cancer (Singh and Krishan, 2019[308]; Ungar et al., 2000[338]). The SASP, a mixture of pro-inflammatory and tissue remodeling factors released by senescent cells, amplifies chronic inflammatory responses and may cause fibrosis, contributing to kidney structural and functional decay (Abrass, 1990[2]). These secretions can alter the homeostasis in tissues by remodeling the ECM, inducing fibrosis deposition, and promoting chemotaxis of the immune cells to the inflammatory site where the inflammatory response is maintained, and further tissue damage continues to occur (Andrade et al., 2018[14]; Lindeman and Goldman, 1986[213]). Chronic exposure to SASP factors exacerbates the progression of CKD and other renal pathologies linked to aging (Glassock et al., 2017[125]). In this context, inhibition against SASP components or their signaling pathways has recently emerged as a potential therapeutic strategy to alleviate the adverse consequences of cellular senescence that are expected to rescue declining kidney function and restore the health span of elderly populations (Shiels et al., 2017[305]; Shu et al., 2015[306]; Yin et al., 2023[378]) (Figure 2(Fig. 2)).

## SIRT1: A Key Regulator of Oxidative Stress and Senescence

### Overview of SIRT1 and its functions

SIRT1 is a novel member of the sirtuin family of NAD+-dependent class III deacetylases that has been identified as a critical regulator of mammalian cellular metabolism and longevity (Cui et al., 2022[82]; Jalgaonkar et al., 2022[160]). SIRT1 is an evolutionarily conserved mammalian protein with a wide range of cellular functions related to metabolism, stress response, and aging processes (Liu et al., 2022[221]; Shen et al., 2021[301]). It mainly deacetylates multiple protein targets and controls their activity, stability and association with other molecules (Chen et al., 2021[74]; Shen et al., 2024[303]). SIRT1 has roles in the complete scheme of metabolic regulation (Tang, 2016[316]). It also deacetylates and activates metabolic regulators like PGC-1α, increasing mitochondrial biogenesis and function and FOXO transcription factors involved in stress resistance and longevity (D'Onofrio et al., 2018[97]; You and Liang, 2023[380]). SIRT1 regulates these pathways to increase cellular energy utilization efficiency and protect cells from stress-induced death (Chang and Guarente, 2014[65]). SIRT1 also maintains genomic stability (Chen et al., 2021[70]). By deacetylating histones and other proteins involved in DNA repair, SIRT6 helps the cell to repair DNA double-strand breaks and maintain chromosomal stability (Chen et al., 2020[68]; Kim et al., 2022[185]). This function is most critical in aging cells as DNA damage is not just associated with nuclear blebbing and a decrease in PTR but also leads to cellular dysfunction and senescence (Alves-Fernandes and Jasiulionis, 2019[12]; Wang et al., 2021[352]). SIRT1 has roles in metabolism, including effects on the genome, but it is also a potent modulator of inflammation (Singh and Ubaid, 2020[309]). It deacetylates the subunit p65 of the NF-κB, a fundamental transcription factor in inflammatory responses, impairing the expression of pro-inflammatory cytokines (DiNicolantonio et al., 2022[93]; Yang et al., 2022[373]). This intervention helps avoid the chronic inflammation purported to be central to aging and age-related diseases (Lu et al., 2023[224]). In the kidneys, SIRT1 inhibits oxidative stress and fibrosis (Kauppinen et al., 2013[175]). It controls the activity of fibrotic factors, including TGF-β and SMAD3, contributing to a protective mechanism against renal aging and disease (Garcia-Peterson and Li, 2021[120]; Patra et al., 2023[267]). Thus, defining the role of SIRT1 presents an essential axis for both targeting and understanding the therapeutic potential against age-induced kidney disease and possibly healthy aging (Meng et al., 2017[233]).

One pathway to SIRT1 expression involves progressing from ROS to cellular senescence and aging (Byrnes et al., 2022[58]). Elevated levels of ROS are known to induce oxidative stress, which can result in cellular damage and trigger a senescence response (Feng et al., 2024[109]). This cellular senescence is characterized by a stable cell cycle arrest and the secretion of pro-inflammatory cytokines, collectively known as the SASP. As cells age and accumulate damage, the expression of SIRT1 is upregulated as a protective mechanism to counteract ROS's deleterious effects and promote cellular repair and longevity (Xu et al., 2019[370]; Yu et al., 2018[381]). In addition to the direct progression from ROS to aging, alternative pathways such as ROS-induced epigenetic modifications can also lead to the expression of SIRT1. ROS can influence various epigenetic marks, including DNA methylation, histone modifications, and non-coding RNA expression (Wang et al., 2014[353]). These epigenetic changes can alter the transcriptional landscape of the cell, leading to the activation of genes involved in stress response and survival, including SIRT1 (Tang et al., 2021[318]; Thirupathi and de Souza, 2017[329]). ROS-induced histone acetylation changes can create a more accessible chromatin state, facilitating the binding of transcription factors that promote SIRT1 expression (Suntar et al., 2020[315]). Furthermore, non-coding RNAs such as miRNAs and lncRNAs can modulate the stability and translation of SIRT1 mRNA, thereby influencing its protein levels in response to oxidative stress (Schottlender et al., 2021[298]; Singh and Ubaid, 2020[309]).

### SIRT1 and oxidative stress regulation

SIRT1 is associated with regulating oxidative stress, a central mechanism of cellular aging and a requirement of many diseases like the kidneys (Beyfuss and Hood, 2018[40]; Cui et al., 2022[82]). SIRT1 modulates the activity of several transcription factors and enzymes implicated in the cellular antioxidant defense system (Wu et al., 2022[367]). SIRT1 is one of those main factors affecting the balance of response to oxidative stress primarily to the transcription factor FOXO3a (Jalgaonkar et al., 2022[160]; Shen et al., 2021[301]). Therefore, SIRT1-deacetylated-FOXO3a in the cell nucleus can be involved in the expression of antioxidant enzymes, including SOD and catalase (Zhang et al., 2017[389]). They are key antioxidant enzymes detoxifying ROS and protecting cells from oxidative stress (Meng et al., 2020[232]). More interestingly, the PGC-1α a coactivator that promotes the biogenesis and function of mitochondria, is one of the SIRT1 substrates (D'Onofrio et al., 2018[97]) since improved mitochondria efficacy results in less ROS creation. A large part of these ROS results in the lowering of the standard of pro-oxidants, which manifest themselves in this phenomenon (Singh and Ubaid, 2020[309]; Xu et al., 2019[370]). SIRT1 binding at chromatin represses key pro-inflammatory NF-κB transcription factors via deacetylation of the p65 subunit, thereby decreasing transcription of pro-inflammatory genes that would otherwise compromise redox homeostasis and harm tissues (Chen et al., 2013[75]; Kong et al., 2017[191]; Shi et al., 2023[304]). This is significant in kidney health as the positive regulatory actions of SIRT1 (Jin et al., 2023[166]). In addition, by increasing antioxidant defenses and attenuating inflammation, SIRT1 performs an antioxidant function. It thereby diminishes oxidative stress, which is a major driving force of renal problems due to aging as well as kidney diseases (DiNicolantonio et al., 2022[93]; Fang et al., 2022[105]; Feng et al., 2021[108]). The function of SIRT1 in controlling oxidative stress provides novel perspectives on therapeutic intervention to safeguard the kidney and enhance healthy aging (Alam et al., 2021[7]; Winiarska et al., 2021[365]).

#### Modulation of antioxidant defenses

SIRT1 enhances antioxidant defenses, which is crucial in reducing oxidative stress and maintaining cellular health by activation of transcription factors which deacetylate them (Halliwell, 2024[136]; van der Pol et al., 2019[341]). Deacetylated form of FOXO3a induces the expression of antioxidant enzymes, such as SOD and catalase, which serve to reduce ROS concentrations (Forman and Zhang, 2021[113]). Activation of PGC-1α induces mitochondrial biogenesis and function, decreasing ROS production and improving mitochondrial efficiency (Bhatt et al., 2020[47]; Bjørklund et al., 2022[49]; Ma, 2013[227]). In parallel, SIRT1 blocks pro-inflammatory pathways, likely in part via deacetylation of the NF-κB p65 subunit, suppressing pro-inflammatory cytokine production that could promote oxidative damage (Arulselvan et al., 2016[17]; He et al., 2023[142]). SIRT1 also activates the cellular antioxidant defense system, which appears crucial for preventing oxidative stress-induced cellular damage and aging (Barnes, 2020[28]; Sies, 1997[307]).

#### Regulation of oxidative stress-related signaling pathways

SIRT1 affects oxidative stress by signaling pathways, mediating oxidative stress, and affecting cell survival (Liguori et al., 2018[208]). It deacetylates FOXO3a and PGC-1α, two pivotal transcription factors (do Nascimento et al., 2008[95]). The expression of antioxidant enzyme SOD, catalase, and other genes that neutralize ROS is enhanced by deacetylated FOXO3a (Pingitore et al., 2015[271]; Zeng and Lu, 2018[386]). PGC-1α activation improves mitochondrial function, decreasing ROS generation through ameliorating mitochondrial efficiency and biogenesis (Agarwal et al., 2012[4]; Katz et al., 2011[174]). SIRT1 also deacetylates the p65 subunit, modulating the NF-κB pathway and decreasing the expression of pro-inflammatory cytokines (Demirci-Çekiç et al., 2022[88]). This combats chronic inflammation and the toll on the system when oxidative stress increases (Chen et al., 2009[73]). These regulatory pathways maintain cellular redox homeostasis, which is required for the resistance to oxidant stress and the extension of cellular lifespan by SIRT1 (Stocker, 2016[313]).

### SIRT1 and cellular senescence modulation

SIRT1 is a notable regulator of pathways linked to cellular senescence, an indispensable stress-response program that causes cells to exit the cell cycle permanently and which is thought to contribute to aging and a spectrum of age-associated diseases (Barnes et al., 2019[29]; Salminen and Kaarniranta, 2012[291]). Aging is most likely initiated by many stimulants, the most significant being oxidative stress, DNA impairment, and the thickening of telomeres, which leads to cellular senescence (Pyo et al., 2020[278]; Yao and Rahman, 2012[376]). Senescent cells that are kept in the body will increasingly become more inert and secrete a series of pro-inflammatory cytokines, chemokines and proteases known as the SASP, which ultimately might contribute to tissue function decline and chronic inflammation (Chung et al., 2010[79]; Hwang et al., 2013[156]). SIRT1 regulates cellular mechanisms of senescence through multiple pathways (Chen et al., 2020[68]). One of the major pathways consists of the deacetylation and, thereby, activation of the tumor suppressive protein p53 (Hekmatimoghaddam et al., 2017[143]; Yi and Luo, 2010[377]). Active p53 can then cause a cell to G2 arrest and undergo apoptosis in response to DNA damage (D'Onofrio et al., 2018[97]). In contrast, SIRT1 deacetylation of p53 leads to SIRT1 facilitating cell survival and DNA repair. Still, without the unwanted senescence characteristic of hyperacetylation-treated cells, it prevents the build-up of senescent cells and the preservation of tissue homeostasis (Lagunas-Rangel, 2022[197]; Lee et al., 2019[200]). SIRT1 also interacts with and deacetylates other components of the cellular senescence process, such as the FOXO family of transcription factors (Kida and Goligorsky, 2016[179]). The FOXO proteins are essential regulators of lifespan and stress resistance. SIRT1 enhances the transcriptional activity of FOXO through their physical interaction and deacetylation of FOXO, leading to up-regulating FOXO-dependent genes associated with the reduction in cellular senescence, such as DNA repair, antioxidant defense and cell cycle control (Cui et al., 2022[82]; Fukuda et al., 2020[117]).

SIRT1 also impacts the NF-κB signaling pathway, which plays important role in inflammation and senescence formation (Guo et al., 2020[133]). As a result of deacetylation of the p65 subunit of NF-κB, transcription of pro-inflammatory genes is lowered by SIRT1, reducing the inflammation that can induce senescence (Poulose and Raju, 2014[274]). In the context of renal aging, modulation of senescence is especially pronounced for SIRT1. Accumulation of senescent cells in the kidney contributes to fibrosis, reduced regenerative capacity, and loss of function (Ala and Ala, 2021[6]). SIRT1 is an important regulator of renal function with protective roles in delaying the pathogenesis and progression of age-associated kidney diseases through improved DNA repair, reduction in oxidative stress, and modulation of inflammatory responses (Wang et al., 2021[356]; Yacoub et al., 2014[371]). Therefore, the current review aims to provide insights into the outcomes of SIRT1 activity modulation on cellular senescence and its underlying molecular mechanism as the regulators of cellular senescence, which promise prospective potential therapeutic options to counteract against aging process and extend health span, especially in age-vulnerable organs such as the kidney (Famulski and Halloran, 2005[103]; Lamb et al., 2003[198]; Maertens and Van Den Noortgate, 2008[228]).

## SIRT1 as a Potential Therapeutic Target in Kidney Aging

### Experimental evidence for SIRT1 activation in kidney aging

Experimental evidence highlights a potential role of SIRT1 in kidney aging, recent studies have demonstrated protection against various kidney age-related changes by activating SIRT1 (Guo et al., 2020[133]; Kitai et al., 2021[189]). Studies on animal models have shown that overexpression of SIRT1 in the kidneys ameliorated age-related renal dysfunction (Kume et al., 2013[194]). For example, transgenic mice with SIRT1 overexpression show less oxidative stress, fibrosis, and improved renal function than their wild-type littermates (Tanriover et al., 2023[319]). Drugs that activate SIRT1, such as resveratrol, have also achieved positive outcomes (Wang et al., 2018[355]). Conversely, in aged mice, resveratrol treatment enhances SIRT1 enzymatic activity, promoting antioxidant enzyme expression, reducing inflammation and protecting against renal fibrosis (Kanbay et al., 2021[171]; Yacoub et al., 2014[371]). These effects together favour the maintenance of kidney integrity and function (Jin et al., 2024[165]). In addition, *in vitro* culture studies with renal cells have shown that SIRT1 activation improves cellular resistance to oxidative stress and delays the onset of cell senescence (Guan and Hao, 2016[130]). Together, these results suggest that it may be therapeutically advantageous to manipulate SIRT1 to mitigate age-related kidney decay and maintain aging kidney health. Further understanding these mechanisms could open new avenues for interventions in age-associated kidney diseases (Meng et al., 2017[233]; Qi et al., 2022[279]).

#### Animal studies

Experimental evidence in animal models shows that activation of SIRT1 attenuates aged-related kidney injury (Yan et al., 2022[372]). Activation of SIRT1 minimizes oxidative stress, inflammation, fibrosis, and renal function improvement (Kitada et al., 2013[187]). Such studies in aged mice reveal it improved kidney structure and function upon SIRT1 overexpression, suggesting its protecting role in renal aging and associated pathologies (Ogura et al., 2021[262]). D-galactose is a monosaccharide; a simple sugar. D-galactose has a history in cellular metabolism and aging analyses (Azman and Zakaria, 2019[21]). High intake induces oxidative stress and age-related damage in animal studies (Wang et al., 2022[354]). Fang et al. reported that empagliflozin attenuates renal senescence in D-galactose-induced aging mice via SIRT1 and oxidative stress reduction.

Conversely, treatment of empagliflozin reversed the changes of BTBD9 and alleviated age-related kidney injury as well as upregulating SIRT1, SOD1, and SOD2. Similarly, *in vitro* empagliflozin exerted protection effects at the cellular level, whereas a SIRT1 inhibitor decreased these effects. These findings demonstrate the ability of empagliflozin to alleviate renal aging by concentrating on SIRT1-related oxidative stress (Fang et al., 2023[106]). Nur77 is an orphan nuclear receptor that participates in multiple cellular functions, such as apoptosis, inflammation, and metabolism (Lith and de Vries, 2021[214]). It is closely related to immune regulation, cancer formation and metabolic diseases (Liu et al., 2021[218]; Zhang, 2007[390]). Yu et al. showed that oxidative stress inhibits the Nur77-SIRT1 axis to reduce SIRT1 expression levels, thereby accelerating aging. The overexpression of Nur77 stabilizes SIRT1, indicating that Nur77 regulates protein expression and renal insulin sensitivity in the aged kidney. Nur77 depletion exacerbated age-related nephropathy, demonstrating the importance of Nur77-SIRT1 signaling in kidney aging and functions. This work provides a novel understanding of oxidative stress and SIRT1 regulation at the molecular level (Yu et al., 2023[382]). 

**Figure 3:**
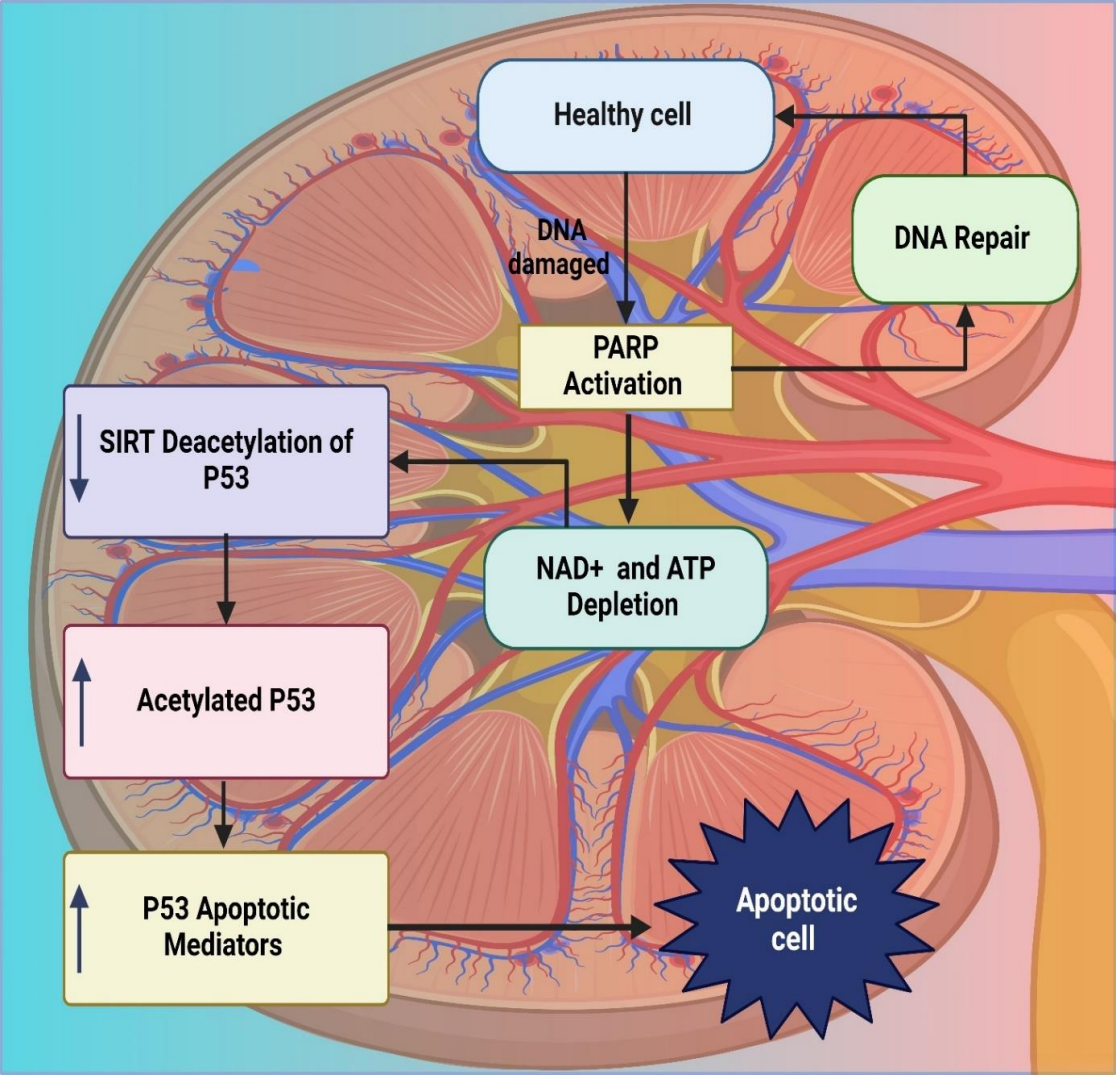
The image illustrates the pathway of cell apoptosis in aging and aging-associated kidney disorders, involving DNA damage and PARP activation, leading to NAD+ and ATP depletion. SIRT deacetylates P53, regulating apoptotic mediators, while DNA repair mechanisms attempt to restore cell health, preventing apoptosis.

IUGR is a condition in which a fetus does not grow at the expected rate during pregnancy. This can then cause low birth weight, birth complications and lifelong health problems (Longo et al., 2014[223]). This may be due to placenta issues, mother complications, or genetics (Darendeliler, 2019[83]). Keshavjee et al. assessed whether suppression of SIRT1 expression and oxidative stress contribute to, a nutshell, improved NAFLD. We studied SIPS using an IUGR rat model. The study revealed pronounced oxidative stress and senescence markers in IUGR males, which may provide a potential pathway to metabolic disease. This work further highlights the repercussions of early life stress into adulthood kidney health (Keshavjee et al., 2022[177]). NAD+, an important coenzyme required for cellular metabolism, is an essential coenzyme required for DNA repair and energy production (Yoshino et al., 2018[379]). A central player in redox reactions involves moving electrons from one molecule to another. Decreasing cellular function and health as NAD+ levels drop with age (Covarrubias et al., 2021[81]; Imai and Guarente, 2014[157]). Braidy et al. demonstrated that NAD+ levels and SIRT1 activity declined as Wistar rats aged in all studied organs, related to an augmentation of oxidative stress and a decrease in mitochondrial function. Reduced SIRT1 activity was accompanied by heightened DNA damage and protein carbonylation. Our study illustrates the potential benefits of preserving NAD+ and SIRT1 levels in protecting against oxidative damage and suggests its therapeutic relevance to aging and aging-associated disorders (Braidy et al., 2011[54]) (Figure 3(Fig. 3)).

Glomerular endothelial cells are important in blood filtration, filtration barrier maintenance, and glomerular structure (Benzing and Salant, 2021[37]; Bose and Cattran, 2014[51]). Chuang et al. demonstrated that the podocyte-specific deletion of SIRT1 in aging mice exacerbated kidney injury, manifested by worsened glomerulosclerosis, albuminuria, and oxidative stress. The results from this study indicated that decreased SIRT1 expression indeed promoted the higher expression of cellular senescence markers and the weaker activation of major transcription factors, which consequently suggested that SIRT1 was essential to protect podocytes from age-related injury and oxidative stress, unveiling its therapeutic potential in this context (Chuang et al., 2017[78]). Cadmium is a toxic heavy metal present as a pollutant in the environment mainly due to industrial activities that include mining and smelting (Peana et al., 2022[268]). This builds up in an individual's organs, destroying them and leading to severe health problems such as kidney failure, bone brittleness and cancer (Genchi et al., 2020[123]; Satarug et al., 2010[293]). Dong et al. *in vivo* experiments were carried out to detect the potential role played by SIRT1 in CKD. The results showed that Cd accelerated the process of renal senescence, fibrosis, and dedifferentiation, which NAC effectively attenuated through the SIRT1-P53 pathway. NAC reduced oxidative stress and reversed senescence, attenuating kidney damage associated with CKD progression. The research indicates that the SIRT1-P53 pathway may offer a therapeutic target in cadmium-induced CKD (Dong et al., 2023[96]). The platinum-based chemotherapy drug is cisplatin, which helps treat testicular, ovarian, bladder, and lung cancers (Leal and García-Perdomo, 2019[199]). It does this by creating damage in the DNA of cancer cells, eventually leading to cancer cell death. It has multiple adverse effects, like nephrotoxicity, ototoxicity, and gastrointestinal disturbances (Rossi and Di Maio, 2016[287]; Xian et al., 2021[368]). Li et al., in a murine model, found that cisplatin caused kidney senescence and fibrosis prematurely. Administration of NAC significantly suppressed this adverse effect by promoting SIRT1 activation and the deacetylation of p53. The findings of this study suggest that SIRT1 serves a protective role against cisplatin-induced renal injury and that SIRT1 activation is a potential target for the prevention of renal dysfunction chronicity after AKI and p53 deacetylation (Li et al., 2019[201]). Hasegawa et al. overexpressed SIRT1 in mice, specifically in kidneys, and created transgenic mice, which protected SIRT1 from cisplatin-induced AKI through peroxisome quality maintenance. Renal tubular apoptosis was diminished by SIRT1 overexpression, which was associated with decreased reactive oxygen species, peroxisome number, and function preservation. This study demonstrates that SIRT1 might have a role in maintaining peroxisomes and protecting against AKI, a plausible mechanism that implicates SIRT1 as a therapeutic target for renal protection (Hasegawa et al., 2010[139]).

Similarly, Lim et al. explored the state and fate of the kidney in aged mice beyond what was known from similar experiments on a background of atherosclerosis. Accordingly, the study showed that the kidneys of older mice exhibit oxidative stress and lower SIRT1, ERR-1α, PGC-1α, PPARα expression, and reduced klotho, an antiaging factor. These changes were accompanied by upregulated albuminuria, mesangial volume, and tubulointerstitial fibrosis. These results indicate that approaches to modulate SIRT1 and its downstream signaling may be beneficial in reducing oxidative stress and pathophysiological changes in the aging kidney (Lim et al., 2012[209]). 

Chronic rising blood pressure can cause hypertensive kidney injury, which arises from damaging blood vessels by the raised blood pressure for so long that it injures the nephrons and leads to the kidney functioning less efficiently (Messerer et al., 2021[234]; Radi, 2019[282]), which can easily lead to chronic kidney disease and can lead to proteinuria, glomerulosclerosis, renal failure. All the more reason for early intervention (Poston and Koyner, 2019[273]). Pushpakumar et al. studied hypertensive kidney injury in aging and reported that oxidative stress and hypermethylation of antioxidant enzymes amplified kidney damage. In aged kidneys, expression of SIRT1, SODs and catalase was decreased and further decreased in ang-II treatment. The results reveal that epigenetic regulation of oxidative stress is fundamentally important in this setting. They imply that it is targeted as a therapeutic approach to ameliorate hypertensive kidney injury with aging (Pushpakumar et al., 2020[277]).

**Figure 4:**
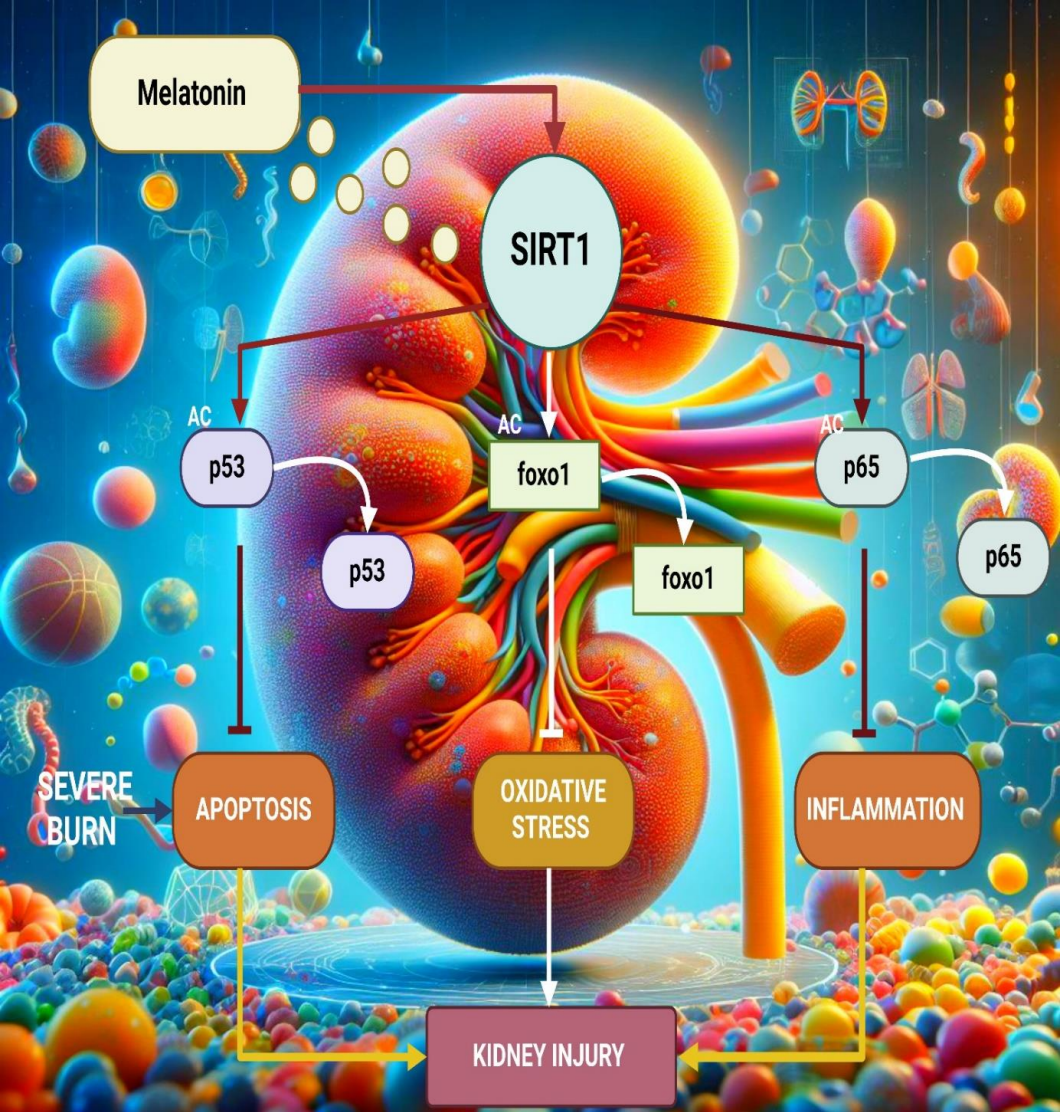
The image illustrates the protective role of SIRT1 in preventing kidney injury from severe burns. Melatonin activates SIRT1, which deacetylates p53 and p65, reducing apoptosis and inflammation. SIRT1 also enhances FOXO1 activity, mitigating oxidative stress and kidney damage.

Furthermore, Bai et al. proved that melatonin protected rats from AKI after severe burns through up-regulating SIRT1. Melatonin significantly attenuated oxidative stress, inflammation and apoptosis in renal tissues. Inhibition of SIRT1 abolished the protective effects, which correlated with enhanced SIRT1 expression. This study reinforces the protective ability of melatonin by activating SIRT1 and supports its clinical application in burn-related kidney damage (Bai et al., 2016[25]) (Figure 4(Fig. 4)).

In another study, Grosjean et al. observed the reversal of glomerulosclerosis in diabetic, sclerosis-prone mice by treatment with FDA-approved drugs (pyridoxamine, enalapril, and pentosan polysulfate) that lower levels of oxidative stress and inflammation. Treatment up-regulated Nrf2, SIRT1, ERα and AGER1 expression, reduced albuminuria and protected against sclerosis. The findings provide significant evidence for an oxidative stress and inflammatory therapeutic target for early diabetic kidney disease protection (Grosjean et al., 2018[129]). The AMPK/PPARα/ UCP2 pathway is important in cellular energy homeostasis and metabolism (Carling, 2017[61]). AMPK activation inhibits PPARα leading to fatty acid oxidation, and UCP2 decreases mitochondrial membrane potential, reducing reactive oxygen species production and increasing energy efficiency (Herzig and Shaw, 2018[144]; Trefts and Shaw, 2021[331]). Rubattu et al. spontaneous hypertensive rat strains demonstrated that the AMPK/PPARα/UCP2 were significantly down-regulated in stroke-prone rats, and this might contribute to the age-associated increase in oxidative stress and inflammation in the brain. These changes were associated with increased vulnerability to hypertension-induced target organ damage. The study demonstrates that this antioxidant pathway is downregulated early in this model, and the data would strongly suggest that this contributes to the vulnerability of the rat to the development of hypertension-induced damage (Rubattu et al., 2015[289]).

Furthermore, Uneda et al. showed that ATRAP-KO mice aged their kidneys faster, developed higher oxidative stress, and had shorter lifespans than their WT mice. The expression of SIRT1 was reduced, and these effects were accompanied by a decreased expression of sirtuin-1. These results collectively indicate that ATRAP may have protective effects against the aging of the kidney via SIRT1-dependent mechanisms, independently of AT1R-signaling, implicating ATRAP as an attractive therapeutic target for the prevention of senescence-driven renal decay (Uneda et al., 2017[337]) (Table 1(Tab. 1); References in Table 1: Bai et al., 2016[25]; Braidy et al., 2011[54]; Chuang et al., 2017[78]; Dong et al., 2023[96]; Fang et al., 2023[106]; Grosjean et al., 2018[129]; Hasegawa et al., 2010[139]; Keshavjee et al., 2022[177]; Li et al., 2019[201]; Lim et al., 2012[209]; Pushpakumar et al., 2020[277]; Rubattu et al., 2015[289]; Uneda et al., 2017[337]; Yu et al., 2023[382]).

#### Human clinical studies 

It was previously demonstrated that human clinical studies reveal that SIRT1 activation has beneficial effects against age-induced renal manifestations through inhibition of oxidative stress and bettering mitochondrial function and autophagy on experimentally induced kidney aging (Afsar et al., 2021[3]; Liu et al., 2015[216]; Poulose and Raju, 2014[274]). Increased kidney SIRT1 is associated with superior renal outcomes and lower expression of senescence markers, supporting it as a potential treatment for aging-related renal pathology (Guo and Bechtel-Walz, 2023[132]). Zbroch et al. measured serum SIRT1 and αKlotho hemodialysis patients with end-stage renal disease. Results showed higher SIRT1 and lower αKlotho levels in hemodialysis patients than healthy controls. The relationship between SIRT1 and αKlotho was dependent on kidney function, considering that high SIRT1 values and low αKlotho levels were associated with reduced kidney function, as established, which suggests their as future novel biomarkers for oxidative stress and cardiovascular disease in renal patients (Zbroch et al., 2020[385]). In another investigation Vlassara et al. studies in diabetic CKD patients have demonstrated that sevelamer carbonate also significantly reduces HbA1c, serum AGEs, triglycerides and markers of inflammation and oxidative stress. This was associated with increased SIRT1 and AGE receptor 1 expression and PMNC TNF levels by this treatment. These results would indicate that the treatment of incipient nephropathy would be interfered with by senility of the gut-derived AGEs of metabolic and inflammatory anomalies and that sevelamer carbonate may be one of the end-stage renal disease-vulnerability factors to retard AGEs (Vlassara et al., 2012[346]).

Similarly, Serrano et al. found that sevelamer carbonate decreased AGEs circulating and cellular in those with type 2 diabetes and diabetic kidney disease, raised antioxidant defenses and reduced pro-oxidants. On the other hand, across the board, the treatment didn't significantly alter HbA1c or albumin/creatinine ratios but did yield benefits for specific subgroups. These findings indicate that sevelamer carbonate may ameliorate oxidative stress and inflammation of DKD, which is worth further confirming (Yubero-Serrano et al., 2015[383]).

### Strategies for SIRT1 modulation 

#### Small molecule activators

Small molecule activators of SIRT1, such as resveratrol and SRT2104, enhance SIRT1 activity, promoting longevity and metabolic health (Panickar and Jewell, 2015[264]). These activators bind to SIRT1, inducing conformational changes that increase deacetylase activity (Sturmlechner et al., 2017[314]). This modulation helps reduce oxidative stress, delay cellular senescence, and improve mitochondrial function, offering therapeutic potential for aging-related diseases (Ungar et al., 2000[338]). SLGT2 inhibitors are a class of drugs used to treat type 2 diabetes. They prevent glucose reabsorption in the kidneys, thereby increasing glucose excretion in urine and reducing blood sugar levels (Escobar et al., 2023[101]).

Additionally, and importantly, SGLT2 inhibitors are beneficial in terms of decreased cardiovascular and renal events beyond their effects on glycemia, rendering them among the preferred options for therapy (Ugusman et al., 2021[336]). Wicik et al. carried out a bioinformatic analysis. They thus identified the SGLT2 interaction network, which singled out SIRT1 as one of the most important interactors and indicated that SGLT2 inhibitors might exert systemic effects via SIRT1 regulation. This analysis uncovered central interactions of SGLT2 with oxidative stress and aging-related proteins, which may indicate the pleiotropic impact of SGLT2 inhibitors that extends beyond glucose lowering (Wicik et al., 2022[362]). Doxorubicin is an anthracycline chemotherapy agent used to treat a variety of cancers, including breast cancer, bladder cancer and lymphoma (Carvalho et al., 2009[62]), by blocking the growth and spread of cancer cells. As an injectable form, it intercalates DNA and inhibits topoisomerase II, thereby generating a free radical which causes DNA double-strand breaks that cannot be repaired. Adverse effects include alopecia, emetesia and cardiomyopathy (Rivankar, 2014[284]; Wu et al., 2022[366]). Xiang et al. showed that fasudil reduced oxidative stress, apoptosis, and senescence in the mouse model of doxorubicin-induced nephrotoxicity with potential involvement of SIRT1 activation. Pathway-directed treatment with Fasudil restored kidney function, DNA damage, and oxidative stress markers. The study implies that fasudil protects against cisplatin nephrotoxicity through oxidative stress and cell senescence (Xiang et al., 2021[369]). 

PPARα agonists are substances activating the PPARα receptor, increasing lipid metabolism, decreasing inflammation, and increasing fatty acid oxidation (Bougarne et al., 2018[53]). They are most commonly employed in treating a range of conditions associated with hyperlipidemia and metabolic syndrome (Janani and Ranjitha Kumari, 2015[161]; Montaigne et al., 2021[243]). Kim et al. showed that the PPARα agonist fenofibrate prevents aging-related decline in renal function, proteinuria, inflammation, and fibrosis in mice. Results of the study revealed that compounds could activate AMPK and SIRT1 pathways, which could improve oxidative stress and mitochondrial dysfunction. PPARα is activated during aging, which is renal protective, due to the activation of AMPK-SIRT1 signaling, which can delay the aging process (Kim et al., 2016[184]). 

Tropisetron is an antiemetic medicine used to prevent nausea and vomiting caused by cancer chemotherapy, radiation therapy, as well as after surgery. It acts on the serotonin receptors of the brain and the gut (de Bruijn, 1992[86]; Yang and Zhang, 2020[374]). Mirshafa et al. examined the impact of tropisetron on aging-mediated renal injury in the D-galactose-induced mouse model. All these beneficial effects of tropisetron treatment were associated with decreased oxidative stress, reduced mitochondrial dysfunction and inflammation, and induction of SIRT1. Moreover, these effects on tropisetron improved renal histopathology and function with the possibility of SIRT1-modulated renoprotective for preventing renal aging (Mirshafa et al., 2024[240]). Resveratrol is a natural polyphenol found abundantly in grapes, berries, and peanuts (Zhou et al., 2021[393]). They are rich in antioxidants and have been reported to have many health benefits, including anti-aging and its positive impact on cardiovascular health and actions against inflammation (Breuss et al., 2019[55]; Galiniak et al., 2019[119]). Wang et al. studied RSV improved mitochondria function and oxidative damage in rat kidneys following hemorrhage shock. SIRT1 and PGC1-α expression was also enhanced by RSV treatment in parallel with the elevation of antioxidant defenses. RSV supplementation during resuscitation ameliorated mitochondrial respiratory capacity impairment. It reduced ROS and lipid peroxidation, which may play key roles in its AKI protection, in conjunction with the improvement of mitochondrial functions and decrease of oxidative stress, as evidenced by the present study (Wang et al., 2015[351]). Pyridoxamine is a form of vitamin B6 having potential protective therapeutic profiles (Giannoukakis, 2005[124]). It serves mainly as an antioxidant, fighting against harmful free radicals and preventing the formation of AGEs (Voziyan and Hudson, 2005[347]). It is currently being studied for diabetes complications and kidney diseases (Voziyan and Hudson, 2005[348]). Simon et al. observed that pyridoxamine treatment in aged female mice increased glomerular SIRT1, ERα, and AGER1 expression and reduced TGFβ expression and collagen deposition. This study demonstrates that pyridoxamine can attenuate age-related oxidant stress and not only age-related kidney damage but perhaps AGE accumulation and, therefore, may be a beneficial therapeutic agent in preventing renal aging (Pereira-Simon et al., 2016[269]). 

#### Natural compounds and nutraceuticals

Modulation of SIRT1 activity with natural compounds and nutraceuticals, such as resveratrol, is a powerful strategy, similar to other compounds such as quercetin, curcumin, and pterostilbene which also activates SIRT1, further promoting lifespan extension and anti-age-related diseases (Du et al., 2022[98]; Zhu et al., 2020[397]). They are adrenal cortex hormones, more commonly known as corticosteroids in the meal, used in diets to take advantage of their anti-inflammatory properties (Liu et al., 2023[219]; Tu et al., 2016[332]). Trehalose is sugar that consists of two glucose molecules and is limited to two glucose molecules. It acts as a power pack and a stress shield in different life forms (Vanaporn and Titball, 2020[342]). Trehalose: A sugar well known to stabilize proteins and cells in a dry state or under heat stress (Chen et al., 2022[67]). Bahri et al. demonstrate that aging rats supplemented with trehalose have lowered SIRT1 levels, mitigated oxidative stress inflammation, and improved kidney histopathology, indicating that trehalose could effectively serve as a dietary intervention for renal aging. Trehalose increases antioxidant ability and reduces malondialdehyde. The data suggest that modulation of SIRT1 by trehalose could attenuate at least some features of age-induced kidney injury (Bahri et al., 2021[24]). 

Nrf2 is a transcription factor that, through a basic leucine zipper motif, regulates the genes that help in the protection of redox homeostasis and assists organisms in the prevention of tissue damage initiated by ROS through the upregulation of antioxidant proteins (Baird and Yamamoto, 2020[26]; He et al., 2020[141]). It is key in cellular defense, decreasing inflammation and detoxification mechanisms (Bellezza et al., 2018[35]). Kim et al. identified resveratrol, an Nrf2 activator, suppressed SIRT1/Nrf2 signaling, alleviated oxidative stress and mitochondrial dysfunction, and attenuated aging-related renal injury in mice. Resveratrol treatment improved renal function, reduced proteinuria, and attenuated age-associated pathophysiologic changes. The current study highlighted the role of resveratrol as an inducer of SIRT1 in attenuating oxidative stress and mitochondrial dysfunction in aging kidneys (Kim et al., 2018[183]). Ellagic acid is a natural plant polyphenol shown to scavenge oxygen-derived free radicals with a potential protective effect on mitochondria (Zhu et al., 2022[395]). Almost as focused on its antioxidant energy, it helps to fight free radicals - thereby decreasing oxidative force and infection (Shakeri et al., 2018[299]). This chemical can also be an anti-cancer, anti-inflammatory, and cardioprotective agent, thus becoming an important dietary constituent concerning human health (Deepika and Maurya, 2022[87]). Naghibi et al. showed that ellagic acid increased SIRT1 and NRF2 in elderly ventral kidneys, decreased oxidative stress, and improved renal function and histopathology. In conclusion, ellagic acid caused increases in antioxidative enzymes and decreases in malondialdehyde, an index of lipid peroxidation, were observed. As conclusion, these results indicate that ellagic acid reduces the susceptibility of the aging kidneys to damage by attenuating local oxidative stress to activate SIRT1 and NRF2 signaling, thus affirming the potential therapeutic agent for renal aging (Naghibi et al., 2023[251]). 

Astragalus membranes contain numerous bioactive components, and APS is one of the foremost effective ingredients (Li et al., 2022[202]). Due to their immunomodulatory antioxidant, antioxidant and anti-inflammatory properties, ticks APS can be used in therapeutic aspects of supporting immune function, reducing oxidative stress and anti-inflammatory diseases (Li et al., 2023[205]; Zhang et al., 2019[391]). Miao et al. demonstrated that Astragalus polysaccharides could alleviate aortic endothelial senescence in aged rats by regulating SIRT1 and downstream p21, p53, and P16. APS treatment improved vascular function and decreased oxidative stress, possibly reflecting its anti-aging effects. The results indicated that APS may alleviate endothelial senescence and oxidative stress via the SIRT1/p53 signaling pathway (Bhat et al., 2024[45]; Miao et al., 2024[236]; Thapa et al., 2024[322]). Similarly, Tousian et al. used Alpha-mangostin to address high glucose-induced memory aging in human umbilical vein endothelial cells. Alpha-mangostin administration had similar beneficial effects to metformin regarding its impact on cell viability, oxidative stress, and accruing senescence markers, as shown in the study. These effects were mediated by the SIRT1 pathway, which indicated that alpha-mangostin may protect against vascular dysfunction and cellular senescence in the hyperglycemia state (Tousian et al., 2020[330]). Ginseng, a formidable traditional medicinal plant, has ginsenoside as a bioactive component (Kiefer and Pantuso, 2003[180]). Because of its health characteristics, ginsenoside functions as an anti-inflammatory, antioxidant, and anti-cancer, which improves the immune system, enhances energy, and brings wellness (Chen et al., 2019[72]; Mancuso and Santangelo, 2017[229]). Kim et al. showed the targeting of Akt/FoxO1 pathways and oxidative counterparts as potential issues of ginsenoside Rc in human renal cells. They show that treatment with the Rc increased catalase expression and scavenged reactive species by interacting with CBP and SIRT1. These results indicate that ginsenoside Rc may partially prevent oxidative stress-induced renal injury by regulating FoxO1 and the SIRT1 pathway (Kim et al., 2014[182]). Chlorogenic acid is one of the many natural compounds in coffee, fruits, and vegetables (Naveed et al., 2018[253]). These properties, which include antioxidant and anti-inflammatory properties, can be partly responsible for their potential health benefits, including improved glucose metabolism, weight management, and cardiovascular protection (Miao and Xiang, 2020[235]; Yun et al., 2022[384]). Hada et al. demonstrated that CGA attenuated vascular senescence by activating the Nrf2/HO-1 signaling pathway. Treatment of CGA increased the expression of SIRT1 and eNOS but decreased the protein levels of oxidative stress markers and senescence-associated proteins. This study confirmed that CGA could alleviate H_2_O_2_-induced endothelial cell senescence and suggested that it is a potential exogenous antioxidant against vascular aging. These results imply CGA as a possible medicine against vascular nutrients (Hada et al., 2020[135]). 

Baicalin is a flavonoid isolated from the roots of *Scutellaria baicalensis* (Hu et al., 2021[150]). It has several pharmacological properties, such as anti-inflammatory, antioxidant, and neuroprotective effects (Bajek-Bil et al., 2023[27]). Baicalin has been widely studied for its potential therapeutic effects, particularly for cancer, cardiovascular disease, and neurodegenerative diseases (Wen et al., 2023[361]). Kim et al. stated that the flavonoid baicalin modulated FoxO1 phosphorylation and acetylation, thus promoting antioxidant defenses in aged rat kidneys. The therapeutic effect of Baicalin may inhibit the production of reactive species and increase the catalase expression, which was accomplished by SIRT1 and the PI3K/Akt pathway. Their results indicate that baicalin may inhibit oxidative stress and aging-related damage, and the underlying mechanism may be related to the modulation of the multiple signaling pathways, providing baicalin as a potential anti-aging agent (Kim et al., 2012[181]). Furthermore Zhang et al. showed that resveratrol prevents high-fat diet-mediated renal injury and cellular senescence in mice through regulation of SIRT1. These results suggested the capacity of resveratrol to protect the kidney from obesity-related renal disease by reducing oxidative stress and ameliorating renal histopathology. This study demonstrates the renoprotective effect of SIRT1 activation by resveratrol, suggesting its value as a therapeutic agent for the renal protection (Zhang et al., 2016[388]). Hydrogen sulfide is a colorless poisonous gas that smells like rotten eggs (Aroca and Gotor, 2022[15]; Bhat et al., 2024[42]; Thapa et al., 2023[324]). It is found naturally in volcanic sources, hot springs, and mineral waters. It is generated naturally by the bacterial decomposition of organic residues without oxygen (Dilek et al., 2020[92]; Huang and Xie, 2023[151]). Hou et al. studied the role of H_2_S in aging kidneys and demonstrated that H_2_S levels were lowered, the expression of H_2_S-producing enzymes CSE and CBS were downregulated in the kidneys of old mice H_2_S donor treatment also alleviated oxidative stress, collagen deposition, and induced antioxidant protein expressions. The amelioration of age-related kidney dysfunction by H_2_S was associated with the activation of Nrf2, suggesting that H_2_S supplementation is a potential therapeutic approach to attenuate age-related kidney dysfunction via decreasing oxidative stress and reinforcing the antioxidant defenses (Hou et al., 2016[147]). In another study Niu et al. showed that the consumption of long-term administration of EGCG expanded the life span of healthy rats by ameliorating liver and kidney functions, arresting age-associated inflammation and oxidative stress. The frequency of death and expression of NF-κB decreased significantly, while the expression of SIRT1 and FOXO3a significantly increased. Accordingly, EGCG treatment could postpone death. Based on these results, EGCG may mitigate hepatic and renal injury and potentially mediate inflammatory and oxidative cascades indicative of increase life span (Niu et al., 2013[258]).

### Lifestyle interventions 

Several strategies affect SIRT1 lifestyle interventions, such as exercise, caloric restriction, and an antioxidant-rich diet (Kitada et al., 2017[188]). Exercise increases the SIRT1 function, restores metabolic health, and reduces inflammation. And this thing is that caloric restriction upregulates SIRT1, promoting longevity and cellular repair (Saldanha et al., 2013[290]; Wang et al., 2021[356]). Those polyphenol-rich diets in berries, green tea, and red wine can also increase SIRT1 levels, which helps increase stress resistance and metabolic function (Qiu et al., 2021[281]). Ning et al. showed that short-term CR spared renal intrinsic aging in aged rats, decreasing oxidative damage and autophagic activity levels. CR increased SIRT1 and AMPK, reduced mTOR, increased autophagy, and lowered oxidative stress markers (Bhat et al., 2023[43]; Thapa et al., 2023[326]). Taken together, this study indicates that short-term CR, via the regulations of the main metabolic modulators and through induction of autophagy, is an efficient intervention to delay the progression of renal aging (Ning et al., 2013[257]). In another study, Wang et al. found that SIRT2 could deacetylate FOXO3a upon oxidative stress and caloric restriction. In the kidney and white adipose tissue of these animals, high expression of SIRT2 leads to increased FOXO3a DNA binding and gene expression of the targets SOD2 and catalase, accompanied by a decrease in reactive oxygen species levels. Its result indicates that SIRT2 is a critical player in the oxidative stress resistance and the survival of the cells during caloric restriction (Wang et al., 2007[350]) (Table 2(Tab. 2); References in Table 2: Bahri et al., 2021[24]; Fang et al., 2023[106]; Hada et al., 2020[135]; Kim et al., 2012[181], 2014[182], 2016[184], 2018[183]; Mirshafa et al., 2024[240]; Naghibi et al., 2023[251]; Pereira-Simon et al., 2016[269]; Wang et al., 2015[351]; Xiang et al., 2021[369]). 

### Challenges and limitations of SIRT1-based interventions

Although promising, interventions based on SIRT1 have several challenges and limitations that should be overcome to develop an effective therapy against age-related kidney dysfunction (Casalena et al., 2012[63]; Hao and Haase, 2010[138]). Most importantly, the delivery and bioavailability of SIRT1 activators, including resveratrol, are the major roadblocks to putting SIRT1 activation into clinical use, although preclinical studies suggest their benefit (Clark, 2000[80]). Resveratrol ingestion is characterized by poor bioavailability, associated with its rapid metabolism and elimination, which largely preclude significant clinical impact (Grinyó, 2000[128]; Ungar et al., 2000[339]). Improving the stability and delivery modalities of SIRT1 activators is important for developing effective drugs so that these compounds can reach sufficient therapeutic concentrations in target tissues (Moritz et al., 1997[246]). While this can lead to several side effects, as many of these activators also interact with other enzymes in the sirtuin family, some other compounds show SIRT1 specificity (Thapa et al., 2023[326], 2024[328]). Such non-specific activation may lead to collateral damage, minimizing the therapeutic effect (Bar-Shai et al., 2008[30]; Bertram, 2013[38]). However, developing selective sirtuin activators for SIRT1 that do not bind undesirable off-target interactions is needed for this safety to be adequate (Luckey and Parsa, 2003[225]). In addition, the chronic consequences of pro-longevity pathways, including SIRT1 activation, are incompletely characterized (Krzesinski and Delanaye, 2014[193]). Although short-term studies have shown beneficial effects on renal aging by SIRT1 activation, the longer-term consequences of chronic SIRT1 activation over a long period remain uncertain (Diz, 2008[94]; Weide and Huber, 2011[360]). Although a role for SIRT1 in repressing cellular senescence might be acknowledged, the possibility that sustained SIRT1 activation may be deleterious in the context of cancer progression and other age-related diseases necessitate further long-term studies to be conducted (Denecke et al., 2015[89]; Maruyama et al., 2010[230]; Vernier et al., 1971[344]). Additionally, differing individual responses to SIRT1 activators represents a further obstacle to clinical utility (Passmore et al., 2005[266]). Many factors, including genetic background, concurrent disease and the environment's influence, can impact the efficacy of SIRT1-based therapies (Davison, 1998[85]; Lindeman, 1993[212]). Given the genuine variation in these individual considerations, personalized strategies will be crucial to ensuring that the effectiveness of therapeutic intervention is optimized and risks minimized (Wang et al., 2021[356]). Lastly, translating conclusions from animal models to human studies is not without its symptoms (Campbell et al., 2021[60]). Most of the preclinical research showing the advantages of SIRT1 activation is in animal models (Nguyen and Corvera, 2024[255]). Human physiology and heterogeneity of human diseases together contribute to different consequences than can be seen in pre-clinical models and require robust clinical trials to evaluate the potential efficacy of SIRT1-based interventions in humans (Betjes, 2020[39]; Kitada et al., 2017[188]; Kuro, 2021[196]). While targeting Klotho, SIRT1, or the SIRT1/Klotho axis is very promising for the pharmacotherapy for kidney aging, inevitable issues, including delivery, specificity, long-term affordability, individual variability, and transgenic and translatable approach from rodents to humans will be essential to be overcome (Das et al., 2014[84]; Kashihara et al., 2012[173]; Little, 2011[215]). Extensive further research and development is required to address these restrictions and realize the full therapeutic capabilities of SIRT1 in age-related kidney dysfunction (Bagnasco, 2005[22]; Morrissey and Yango, 2006[247]). 

## Conclusion and Future Perspective

The interplay between oxidative stress and cellular senescence is significant in renal aging, driving functional decline and heightened disease susceptibility in these organs. Among those is the NAD+-dependent deacetylase SIRT1, which appears to be a major player in dampening these harmful processes. SIRT1 regulates the cellular ROS level by upregulating the transcription factors such as FOXO3a, PGC-1α and the antioxidant enzyme, including SOD, catalase, which in turn neutralize the ROS and keep the cellular redox homeostasis. SIRT1 also regulates senescence by deacetylating proteins like p53 and FOXO, activating DNA repair enzymes, lowering inflammation, and boosting stress resistance. SIRT1, a NAD-dependent protein deacetylase, protects against aging-induced renal injury, as *in vitro* and animal experimental evidence shows. 

Further research is also needed to improve the bioavailability and stability of SIRT1 activators through developing more effective delivery systems. Such delivery could be fundamentally revolutionized using advanced drug delivery platforms, including nanotechnology, to target tissues at sustained concentrations that are pharmacologically active. Therefore, discovering selective SIRT1 activators without off-target effects may contribute to their safety and efficacy. Targeting SIRT1 for protective intervention into kidney aging is of great promise. Thus, tackling challenges related to mechanisms of action, delivery, specificity, long-term toxicity, intersubject variability, and translational studies is essential. Further research and development are necessary to fully exploit the benefits of SIRT1 in the context of improved renal health and extend kidney and health span in the aging population.

## Declaration

### Consent for publication 

All authors gave their consent for publication. 

### Competing interests 

No authors have any conflict of interest or competing interests to declare. 

### Funding 

No funding was received to perform this study. 

### Author contributions 

WHA researched the data and wrote the first draft of the manuscript. SSA edited the manuscript. All authors reviewed and approved the final version of the manuscript. Waleed Hassan Almalki is the guarantor of this work.

## Figures and Tables

**Table 1:**
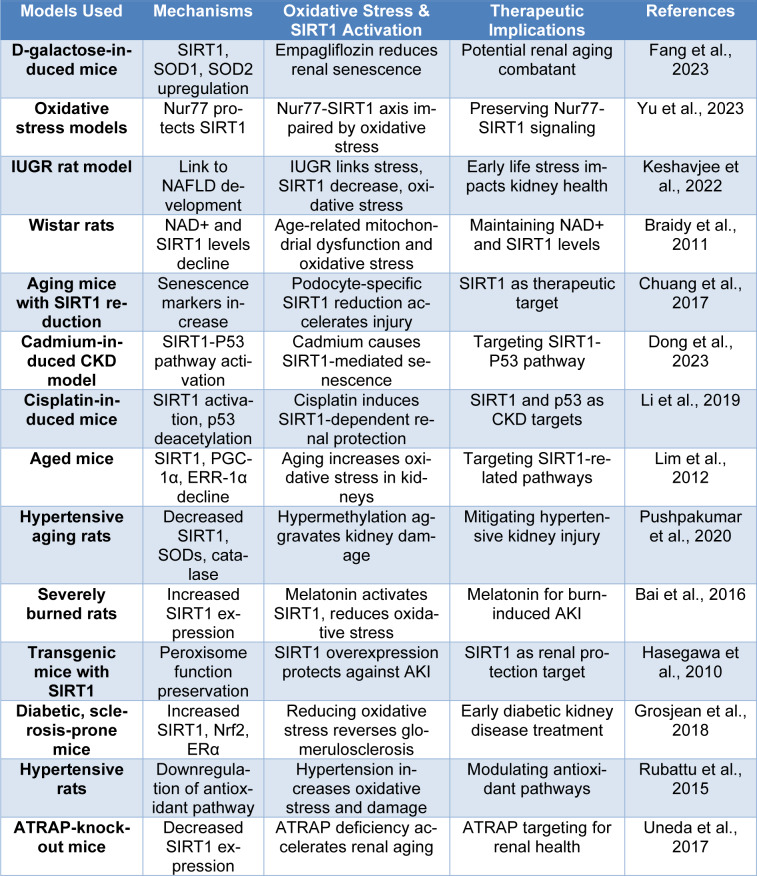
This table summarizes various studies on the role of SIRT1 in renal health, highlighting the models used, mechanisms involved, effects on oxidative stress and SIRT1 activation, and potential therapeutic implications. It provides a concise overview of how targeting SIRT1-related pathways may mitigate kidney aging and disease

**Table 2:**
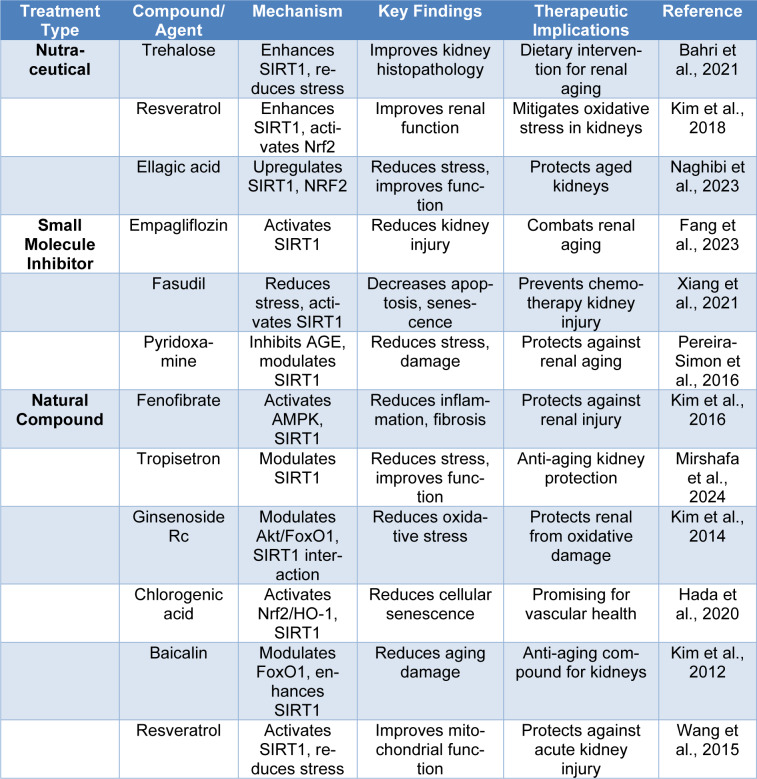
This table summarizes various compounds and agents studied for their effects on renal aging, detailing their mechanisms, key findings, and therapeutic implications. Each entry includes SIRT1 modulation and oxidative stress reduction.

**Figure 1:**
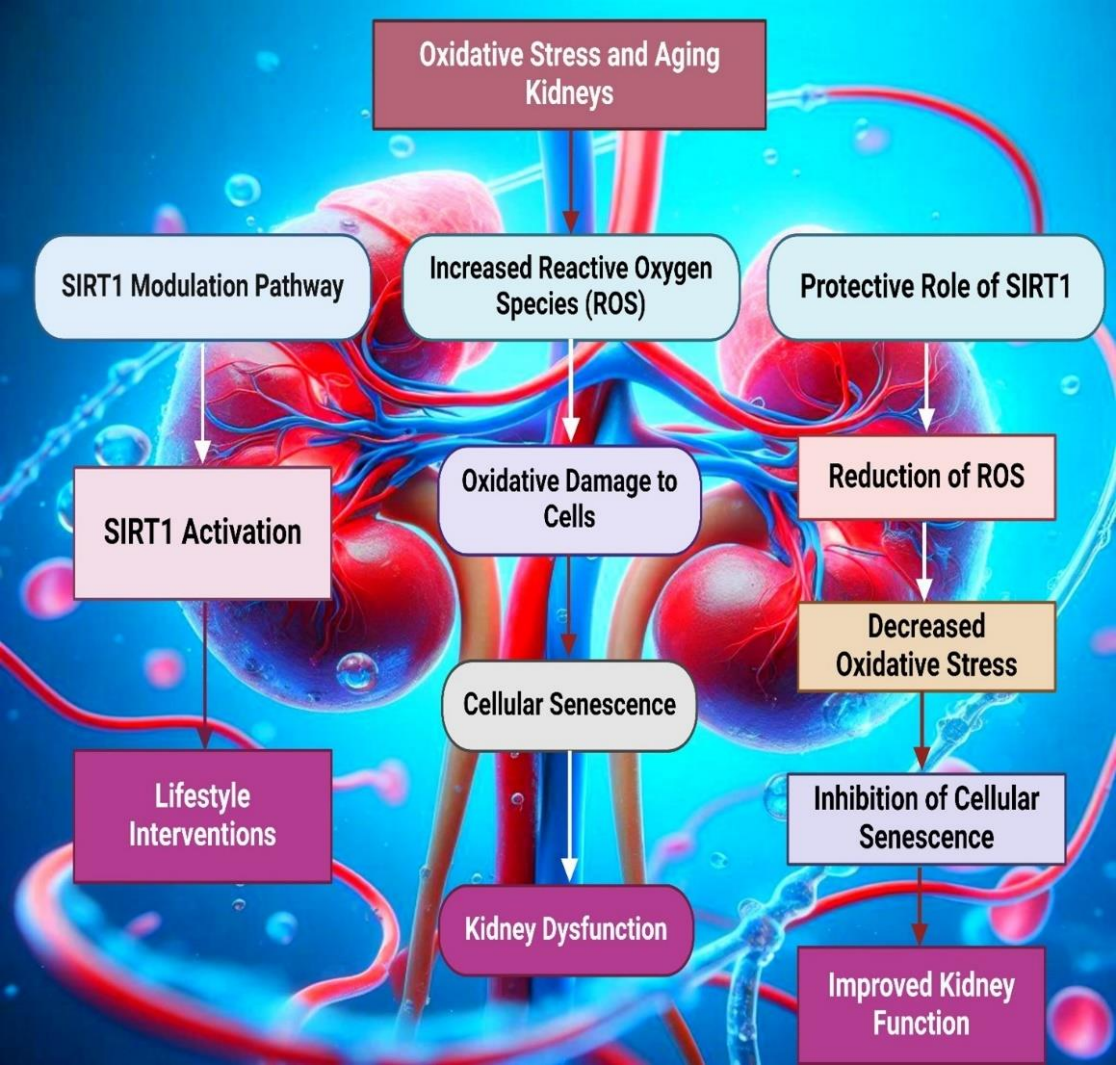
Graphical abstract
